# Somatic Dimorphism in Cercariae of a Bird Schistosome

**DOI:** 10.3390/pathogens11030290

**Published:** 2022-02-24

**Authors:** Miroslava Soldánová, Petra Kundid, Tomáš Scholz, Roar Kristoffersen, Rune Knudsen

**Affiliations:** 1 Institute of Parasitology, Biology Centre of the Czech Academy of Sciences, 370 05 České Budějovice, Czech Republic; petra.kundid@paru.cas.cz (P.K.); tscholz@paru.cas.cz (T.S.); 2 Department of Parasitology, Faculty of Science, University of South Bohemia in České Budějovice, 370 05 České Budějovice, Czech Republic; 3 Department of Arctic and Marine Biology, Faculty of Biosciences, Fisheries and Economics, UiT The Arctic University of Norway, N9037 Tromsø, Norway; roar.kristoffersen@uit.no (R.K.); rune.knudsen@uit.no (R.K.)

**Keywords:** trematodes, cercariae, bird schistosome, *Trichobilharzia*, polymorphism, phenotype

## Abstract

Phenotypic polymorphism is a commonly observed phenomenon in nature, but extremely rare in free-living stages of parasites. We describe a unique case of somatic polymorphism in conspecific cercariae of the bird schistosome *Trichobilharzia* sp. “peregra”, in which two morphs, conspicuously different in their size, were released from a single *Radix balthica* snail. A detailed morphometric analysis that included multiple morphological parameters taken from 105 live and formalin-fixed cercariae isolated from several naturally infected snails provided reliable evidence for a division of all cercariae into two size groups that contained either large or small individuals. Large morph (total body length of 1368 and 1339 μm for live and formalin-fixed samples, respectively) differed significantly nearly in all morphological characteristics compared to small cercariae (total body length of 976 and 898 μm for live and formalin samples, respectively), regardless of the fixation method. Furthermore, we observed that small individuals represent the normal/commonly occurring phenotype in snail populations. The probable causes and consequences of generating an alternative, much larger phenotype in the parasite infrapopulation are discussed in the context of transmission ecology as possible benefits and disadvantages facilitating or preventing the successful completion of the life cycle.

## 1. Introduction

Phenotypic polymorphism is a commonly observed phenomenon in nature throughout the animal kingdom, arising from genetically based differentiation or phenotypic plasticity, or a combination of both (e.g., [1,2,3,4]). It is traditionally defined as an intraspecific variability in observable characteristics of an organism’s trait, implying the co-occurrence of two or more distinct morphs of individuals (i.e., alternative phenotypes) in a population of the same species, inhabiting the same habitat at the same time [5,6]. Such phenotypic variability can be observed across morphological, developmental, behavioral, biochemical, and physiological traits. For instance, conspecific organisms within a given population may differ in body size, growth, reproduction, diet, habitat dispersal and use, and anti-predator behavior, primarily to improve the performance, overall productivity and thus persistence of the population [7]. Probably the best-known and classic examples are polymorphic casts in social insects with clear morphological adaptations to different roles [8] or sexual dimorphism in birds [9]. Many examples of intraspecific polymorphism are also found among uni- and multicellular parasites (e.g., [10,11,12,13,14]), which constitute a substantial proportion of global species diversity [15,16].

Digenean trematodes represent a large and widespread parasitic group of helminths (Platyhelminthes: Neodermata) characterized by complex life cycles that involve sexual reproduction in the definitive host and clonal multiplication (or asexual reproduction by parthenogenesis, see [17]) in the first intermediate molluscan host [18]. The family Schistosomatidae includes mammalian and bird parasites (e.g., species of the most common genera *Schistosoma* and *Trichobilharzia*), which produce eggs that hatch in a first free-living larva— miracidium—in the aquatic environment [19,20]. After successful penetration of the suitable snail host (e.g., [21]), each invading miracidium gives rise to a generation of sporocysts, producing a large number of the second free-living dispersive stages—cercariae that are shed into the environment to infect a definitive host. As all individuals share the same genotype originating from a single miracidium, subsequent developmental stages including cercariae represent genetically identical individuals—clones [17,18]. 

Much of the research on the morphological polymorphism in trematodes has been conducted on adult worms to better differentiate closely related taxa in taxonomic studies in the pre-molecular era (reviewed in [10,22]). The most intriguing example of an extreme somatic polymorphism in clonal trematode larval stages is the existence of reproductive and soldier redial castes differing in morphology and size, in addition to their function, behavior, and site location within the snail body (e.g., [23,24,25]). The use of experimental and/or genotyping molecular methods enabled phenotypic variation studies also in clonal cercariae such as output rates, activity and survival patterns, photo- and geotaxis behavior, or infection success [26,27,28,29,30,31,32]. As mammalian and bird schistosomes serve as model organisms for studying various biological and ecological aspects of parasitism and host–parasite interactions, research on their polymorphism is more advanced than in other trematode groups, focusing on host-induced morphometrical alterations (e.g., [33,34]), genetic diversity of different developmental stages (e.g., [35,36,37,38,39]), or a compatibility polymorphism in snail-schistosome interactions (reviewed in [40,41]). As far as we are aware, only one study to date has detected somatic dimorphism in larval schistosomes (cercariae of *Schistosoma mansoni*) during long-term breeding of the snail host (*Biomphalaria glabrata*) under laboratory conditions [42], but a similar case has not yet been reported for schistosome larvae infecting birds, originating from naturally infected snails. Hence, the presence of polymorphism in genetically identical individuals (clones) represents an interesting, but poorly understood biological phenomenon, which deserves future studies. 

In this study, we report a unique case of somatic dimorphism in cercariae of a bird schistosome species isolated from a single naturally infected snail in a subarctic lake, which shed cercariae markedly differing from each other in body sizes. The aim is to document the existence of size-different cercarial morphs based on their morphometric characterization and comparisons of multiple morphological parameters of conspecific cercariae from a population of field-collected snails. The probable causes and consequences of generating an alternative phenotype in the parasite infrapopulation are discussed in the context of transmission ecology as possible benefits and disadvantages facilitating or preventing the successful completion of the life cycle.

## 2. Results

Molecular identification of the bird schistosome isolates sequenced from the single snail verified that both large and small morphs of cercariae belong to the same species *Trichobilharzia franki* haplotype “peregra” sensu Jouet et al. [43] (hereinafter referred to as *Trichobilharzia* sp. “peregra”, see Materials and Methods). The two sequences were deposited in GenBank under accession numbers OM716986 (small morph) and OM716987 (large morph). 

**Figure 1:**
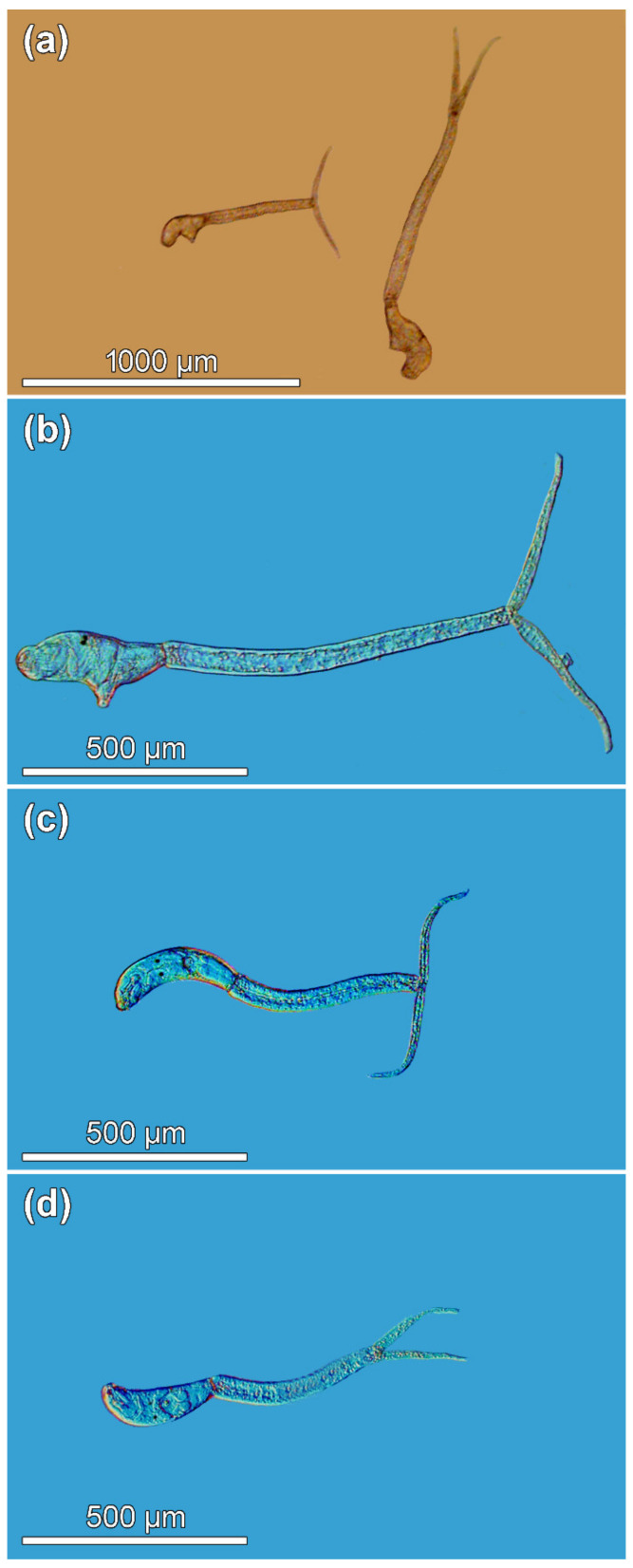
*Trichobilharzia* sp. “peregra”, microphotographs of live cercariae. (**a**) Comparison of two distinct size morphs isolated from a single *Radix balthica* snail. (**b**) Large morph isolated from a single *R*. *balthica* snail individual. (**c**) Small morph isolated from a single *R*. *balthica* snail individual. (**d**) Cercaria representing individuals in the other small group isolated from four *R*. *balthica* snails that were obtained from the same collection in Lake Takvatn, Norway.

Biometric evaluations of 105 cercariae (38 live and 67 formalin-fixed), including the two size-different morphs and other *Trichobilharzia* sp. “peregra” cercariae isolated from naturally infected *Radix balthica* (Linnaeus, 1758) (Gastropoda: Lymnaeidae) within the snail population sampled in September 2013 (Figure 1, Table 1), showed differences in cercarial sizes with respect to fixation method (see Materials and Methods). Higher variability in morphometric data for specimens of live cercariae was due to the contracting and stretching nature of moving live cercariae compared to fixed dead larvae when photographed (Table 1; see also Appendix A Table A1 for metrical data for live specimens, and Table A2 for specimens fixed in hot formalin). 

As for comparisons of cercarial dimensions among the three groups (large and small morphs, and other small cercariae; Table 1), the results of one-way ANOVA (analysis of variance) tests revealed significant differences in the majority of morphometric parameters, regardless of the fixation method (Table 2). 

**Figure 2:**
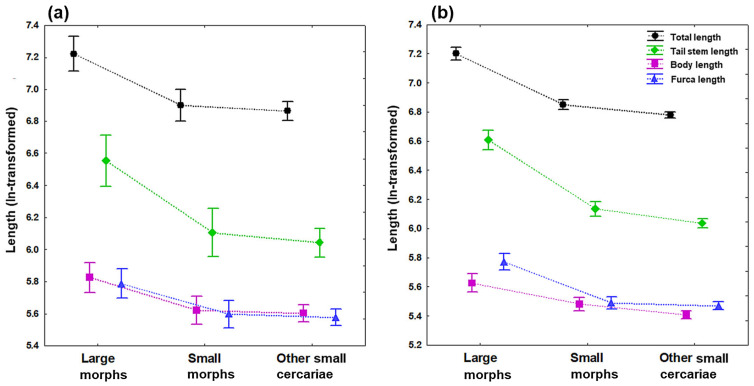
Length of four main body dimensions of cercariae of *Trichobilharzia* sp. “peregra” (differentiated by colors and symbols: total length in black and circles; body length in violet and squares; tail stem length in green and diamonds, furca length in blue and triangles). Measurements are based on (**a**) live specimens and (**b**) formalin-fixed specimens categorized into three groups: large and small morphs isolated from a single *Radix balthica* snail individual, and other small cercariae isolated from four *R*. *balthica* snail individuals (see Table 1 for the number of measured specimens). Symbols denote the mean values and vertical bars 0.95 confidence intervals.

The subsequent pairwise post hoc Tukey’s HSD (honestly significant difference) tests detected considerably larger cercariae in the large group compared to those cercariae in both small size groups (most *p* < 0.05), while there were no significant differences in most measurements between small morphs and other small cercariae (most *p* ˃ 0.05). The most pronounced size differences between large and small cercariae are especially evidenced by four main length parameters (Figure 2), with a considerably greater total length by 374–458 μm (range of mean values), body length by 37–68 μm, tail stem length by 253–320 μm, and furca length by 56–83 μm, depending on the fixation (Table 1, Appendix A Table A1 and Table A2). However, some body dimensions differed from this commonly observed pattern, being variable between live and formalin-fixed specimens (post hoc tests; two and six parameters, respectively). For live specimens, the diameter of acetabulum in large morph was greater than that in small morph only (*p* < 0.05). Simultaneously, small morph had much smaller acetabulum compared to other small cercariae (*p* < 0.05). Furthermore, large cercariae had significantly greater distance between the eye spot and the anterior body end compared to cercariae in the other small group (*p* < 0.01). For formalin-fixed samples, small morphs were significantly longer than other small cercariae in total, body and tail stem length (Figure 2b), and tail stem length to furca length ratio (all *p* < 0.05). Furthermore, significantly greater body width and the distance measured from the eye spot to anterior body end were detected only for large cercariae when compared with other small cercariae (both *p* < 0.001), the latter parameter being also longer in cercariae in the small group than in the other small cercariae (*p* < 0.05).

Small cercariae in our study, especially those isolated from the additional four snails, correspond in their measurements to dimensions of *T. franki* haplotype “peregra” cercariae reported by Jouet et al. [43] (Table 1). However, cercariae in both these small groups differ in the total length about 106–130 μm (range of mean values) for live isolates and about 17–80 μm for formalin-fixed isolates, mainly due to a much longer tail stem and furcae in our samples. The remaining parameters are slightly larger, slightly smaller, or overlapping in measurements, the variation depending on the fixation method (Table 1). 

Overall, the above results for both live and formalin-fixed specimens of *Trichobilharzia* sp. “peregra“ demonstrate that analyzed cercariae, which were initially categorized into three groups based on their origin from snails and size, form just two size categories, one of large individuals (average total body length of 1368 and 1339 μm for live and formalin-fixed samples, respectively) and the second of small individuals (average total body length of 976 and 898 μm for live and formalin samples, respectively).

## 3. Discussion

In the present study, we demonstrated a phenotypic somatic dimorphism in cercariae of the bird schistosome *Trichobilharzia* sp. “peregra” shed from a single lymnaeid *R. balthica* snail individual. To our knowledge, this is a unique polymorphism case reported for cercariae of bird schistosomes, the second case in schistosomes (Pino et al. [42] documented size polymorphism in cercariae of the mammalian *Schistosoma mansoni* shed by laboratory-reared snail hosts) and one of very few reports of this extremely rare phenomenon in trematode larvae (e.g., [23,24,25]).

Our comparative morphometric analysis of clonal cercariae of the same *T*. *franki* haplotype “peregra” sensu Jouet et al. [43] provides reliable evidence for the division of cercariae into two size categories containing either large or small individuals, the former showing significantly higher values for most measured morphological characteristics. While examining patent infections, cercariae of the large morphotype were observed emerging in smaller quantities. This, together with similar dimensions of cercariae of *T*. *franki* haplotype “peregra” published by Jouet et al. [43], although from geographically distant regions (France and Iceland), leads to the assumption that all small individuals produced from a single snail and from the other four snails represent the normal/commonly occurring phenotype in natural snail populations. This is further supported by the fact that only cercariae corresponding to the small morph were always observed in our extensive collection of snails in Lake Takvatn between 2012 and 2019, during which we processed more than 4200 *R*. *balthica* across different seasons and found 85 patent infections with *Trichobilharzia* sp. “peregra” (see subsets of live cercariae of *Trichobilharzia* sp. “peregra” about 940 μm long in Born-Torrijos et al. [44,45]). 

Free-living cercariae are highly important stages in the transmission process between intermediate and definitive hosts [46,47,48], but the probability of reaching the next host is low due to various abiotic and biotic barriers lurking in an aquatic environment (e.g., [49,50]). Like many other trematodes, schistosomes use multiple strategies to increase contact with their bird hosts through cercariae-specific swimming and dispersal behavior, synchronized emergence with peak activity of birds, and responses to host cues [51,52,53,54]. The presence of large cercarial morphs within the bird schistosome infrapopulation may have some additional benefits for the trematode transmission to their definitive host. Firstly, larger individuals likely possess larger penetration glands with a higher volume of histolytic enzymes, which can lead to faster skin penetration and tissue invasion of a bird host. Secondly, large cercariae may remain active longer and thus prolong their survival in the aquatic environment. Cercariae are non-feeding larvae with a lifespan strongly dependent on the non-renewable glycogen reserves, which continuously decreases as cercariae age [55,56]. Glycogen is primarily stored in the main locomotion organ important for swimming and dispersal—the tail [57], which contains about half of its total amount [58]. Large cercariae in our study had a tail slightly more than half as long as small morphs, suggesting a greater quantity of glycogen available to potentially extend cercarial survival. In contrast, larger cercariae generally swim faster by more intense movements [57], which results in a faster depletion of glycogen. Increased survival is directly associated with a longer active period, during which cercariae are most infectious to their hosts within a few hours after their emergence from snail hosts [56,59,60,61]. Thus, somatic dimorphism in cercariae may play an important role in the transmission ecology of trematodes in terms of infection success. 

In contrast, there are also certain disadvantages for the transmission associated with local ecosystem biodiversity in a form of dilution effect of parasite infective stages that leads to a reduction in parasite population size [50,62,63,64,65], including dilution of bird schistosomes [66]. In Lake Takvatn, cercariae of *Trichobilharzia* sp. “peregra” are extensively consumed by invertebrate and vertebrate non-host predators at overall higher consumption rates compared to other trematode taxa [44,45]. The greater vulnerability to predation has been attributed to the dispersion strategy and much larger body of *Trichobilharzia* sp. “peregra” cercariae, as also documented for other predator–prey–parasite interactions (e.g., [67,68,69,70]). Therefore, generating an alternative phenotype, which may benefit from owning a large body potentially facilitating transmission of cercariae, can be at the same time substantially mitigated.

Phenotypic polymorphism may arise from genetic polymorphism, meaning a change in gene expression or use, where the phenotype of each individual is genetically determined, or from phenotypic plasticity, where the phenotype reacts flexibly to environmental stimuli, allowing organisms to adapt and survive in changing environment. A mixed strategy of development combines both mechanisms, where the phenotype is randomly assigned during development, possibly as a result of epigenetic modifications (e.g., [1,2,3,4,71]). 

The effect of phenotypic plasticity on the development of large cercarial morphs of *Trichobilharzia* sp. “peregra” inside a snail individual could have been induced by a host environment (snail size) or an external environment acting on the host (the temperature). The effect of snail size on dimensions of cercariae, when larger snails produced larger cercariae, has been demonstrated in different snail–mammalian schistosome systems [72,73], but no such relationship has been observed for cercariae of three species of *Trichobilharzia* [74]. Moreover, the snail releasing two morphs was of only slightly more than medium size (length × width; 14.2 × 9.2 mm) within a group of all 15 infected snails releasing *Trichobilharzia* sp. “peregra” cercariae (mean length ± SD × mean width ± SD in mm; 12.8 ± 2.4 × 7.8 ± 1.4; range length × range width in mm; 8.2–16.5 × 5.3–10.0). The intramolluscan development of trematodes is strongly temperature-dependent, accelerating with increasing temperature [75]. The prepatent period (i.e., the time before cercariae emerge from a snail host) of bird schistosomes can range from 3 to 10 weeks [53,76] during which larval stages are indirectly exposed to seasonally changing water conditions, which also may potentially result in the formation of an alternative phenotype. However, the less pronounced seasonal fluctuations in water temperature during the parasite’s prepatent period at high latitudes (i.e., 13 °C in August, 8–10 °C in September in Lake Takvatn) compared to temperate ecosystems would likely have a mild effect of these host- and abiotic-related factors. 

Cercariae of a given species are considered to be clones carrying identical genetic information if issued from a single miracidium. However, even these monomiracidial infections may demonstrate a substantial genetic interclonal (i.e., between clones isolated from different snails) and some levels of intraclonal variability (i.e., within clones isolated from a single snail) [35,37,38,77,78]. The latter variability has been suggested to have asexual origin, as it can occur only during parthenogenesis [79]. While this variability could be a possible explanation for the formation of two size morphs *Trichobilharzia* sp. “peregra” found in the present study, differences in phenotypic characteristics of cercariae such as morphology and behavior were found conspicuous at the interclonal level [27,28]. It implies that only one phenotype is likely generated under the scenario of monomiracidial infection. There was a relatively high prevalence of the bird schistosome in our study system (5.7%) compared with other systems (usually less than 1%, [53]) that may indicate multimiracidial infection of single snail releasing two morphs in Lake Takvatn, as noted elsewhere (e.g., [80,81]). Hence, the most probable explanation for occurrence of such conspicuously different morphs is the simultaneous development of two miracidia originating from different bird hosts in the same snail. 

Nevertheless, the aforementioned mechanisms still would not explain the extremely rare frequency in the occurrence of large morphs in schistosomes. More likely, random processes occurring during the development of adult bird schistosomes in definitive bird hosts are the cause of somatic dimorphism rather than genetic polymorphism or phenotypic plasticity. However, it is difficult to draw any definite conclusion, especially given the clear aim of our study and the enormous complexity of internal and external factors influencing trematode development at all life cycle levels. This paper documents the first case of phenotypic polymorphism in clonal cercariae of schistosomes observed in a naturally infected snail host. Future studies should focus on elucidating ecological and evolutionary importance of this unique biological phenomenon that has been rarely documented in trematodes.

## 4. Materials and Methods

### 4.1. Sample Collection and Species Identification

In early September 2013, a total of 280 *R. balthica* were randomly collected by hand in the littoral zone of Lake Takvatn located in northern Norway (69°07′ N, 19°05′ E). Patent (cercarial release) and prepatent (intramolluscan stages—sporocysts) infections with bird schistosomes were examined in laboratory according to Soldánová et al. [82].

A total of 16 snails (5.7%) harbored larvae of bird schistosomes, all but one releasing cercariae. While examining patent infections, two distinct morphotypes of bird schistosome cercariae, which differed conspicuously in body size but seemingly not in their morphology, were observed emerging from a single snail individual (Figure 1a–c). To verify the identity of the larvae (and to eliminate possible double infection) isolated from a single snail individual in our study, cercariae of both size morphs were carefully separated and fixed in molecular grade ethanol for DNA isolation and sequencing. Following the protocol of sequencing analysis of bird schistosomes based on two internal transcribed spacers (ITS1 and ITS2) of the rRNA gene, molecular data revealed that the two newly generated sequences are identical to each other and to sequences for the *T*. *franki* haplotype “peregra” previously found in Lake Takvatn [82], confirming that both cercarial morphotypes belong to the same species. Additionally, four infected *R*. *balthica* individuals were randomly selected to obtain cercariae for morphometric comparison with the two morphs. These cercariae were visually inspected as small individuals (Figure 1d) and identified as *T*. *franki* haplotype “peregra” sensu Jouet et al. [43] based on molecular characterization of specimens from two of these four snails [82] or that cercariae obtained from the remaining two snails come from the same sampling conducted in September 2013 and only one schistosome so far occurred in the lake [44,45,82]. However, given the current knowledge of *Trichobilharzia* systematics with *Trichobilharzia franki* Müller & Kimmig, 1994 parasitizing its type snail host *Radix auricularia* (Linnaeus, 1758) [43,83,84,85], *T*. *franki* haplotype “peregra” in *R*. *balthica* from lake Takvatn represents previously undescribed species [82] and is therefore referred to as *Trichobilharzia* sp. “peregra”.

### 4.2. Morphometric Characterization

Cercariae of *Trichobilharzia* sp. “peregra” produced by the single snail shedding two morphs as well as those obtained from additional four snails were fixed in hot 4% formaldehyde solution (formalin) for metrical characterization and subsequent comparative analyses, in addition to measuring live cercariae. In this study, both live and formalin-fixed cercariae of *Trichobilharzia* sp. “peregra” were used to provide comparative data to describe the present case of somatic dimorphism. A total of 105 cercariae (38 live and 67 formalin-fixed) were photographed with a digital camera of an Olympus BX51 microscope (Olympus Optical Co., Ltd., Tokyo, Japan). Measurements (in micrometers) were then taken from photographs using the program ImageJ [86]. In total, 12 morphometric body characteristics were measured and three ratio parameters were calculated based on body dimensions, i.e., the body length in relation to the body width, body length in relation to the tail stem length, and the tail stem length in relation to the furca length (Table 1). 

**Figure A1:**
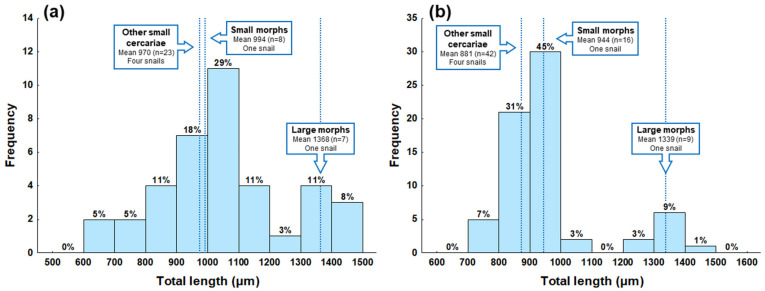
Frequency distribution of cercarial sizes represented by the total length (sum of lengths of body, tail stem, and furca) of *Trichobilharzia* sp. “peregra” isolated from naturally infected *Radix balthica* snails. Measurements are based on (**a**) live specimens and (**b**) formalin-fixed specimens. Boxes represent three groups of cercariae (large and small morphs, and other small cercariae) with data on the mean total length (dotted lines), number of cercariae (*n*), and the origin of cercariae from snail hosts.

To demonstrate the existence of somatic dimorphism in conspecific cercariae, dimensions were compared between the large and small morphs that were released from the same snail individual; dimensions of other cercariae from four other snails were used as a comparative population. Hence, all cercariae were categorized into three groups prior statistical analyses as follows: (i) large morph (isolated from a single snail; Figure 1b), (ii) small morph (isolated from a single snail; Figure 1c), and (iii) other small cercariae (isolated from other four snails; Figure 1d). The material of both live and fixed cercariae in each group originated from the same set of snails. The first visual data inspection indicated two sizes of cercariae comprising either large or small specimens (Figure 1a–d), which was furthermore supported by the bimodal shape of data based on the frequency distribution of cercarial total length (Figure 1 and Appendix A Figure A1). The above-mentioned division of cercariae into three “size” groups was made to avoid bias caused by pooling small cercariae from four additional snails with relatively few cercariae of small morph isolated from the single snail. The ideal scenario would be to use an equal number of specimens in each group, but the snail shedding both size morphs died soon after the parasite’s identification, photographing, and fixation of cercariae. Measurements obtained in our study were compared with dimensions of *T*. *franki* haplotype “peregra” cercariae published by Jouet et al. [43].

To evaluate data statistically, we first tested whether the live and formalin-fixed specimens can be merged together to increase the number of specimens in our dataset. A paired sample *t*-test performed on the total length of cercariae (ln-transformed) was used in analyzing differences between live and fixed material in each corresponding size group. While no significant differences were detected for large morphs (t = 1.163, df = 14, *p* ˃ 0.05), formalin-fixed cercariae were significantly smaller than live specimens in both groups of small cercariae (small morphs, t = 2.312, df = 22, *p* < 0.05; other small cercariae, t = 2.788, df = 63, *p* < 0.01; compare means of total length in Table 1). Therefore, live and formalin-fixed specimens were analyzed separately in a series of univariate comparisons by analyses of variance (one-way ANOVAs) to assess differences in cercarial sizes among the three size groups of cercariae. Post hoc Tukey’s HSD tests were performed to detect differences in individual morphometrical characters among pairs of cercarial groups. Morphometric data of all 12 body characteristics as well as three ratio parameters were used in both data sets, but only the total length and lengths of the body, tail stem, and furca are displayed graphically. As the main goal of this study is to demonstrate the existence of morphological dimorphism between cercarial clones, we believe that the simple comparison based on separate ANOVA analyses, together with four length parameters selected for better illustration, best describe the intraspecific somatic variability in *Trichobilharzia* sp. “peregra” cercariae. All analyses are based on ln-transformed measurements and carried out using Statistica 7.0 software package (StatSoft Inc., Tulsa, OK, USA) with significance levels set at 0.05.

## Figures and Tables

**Table 1:** Comparison of measurements (means in micrometers) of cercariae of *Trichobilharzia* sp. “peregra” among groups of cercariae presented in this study (large and small morphs, and other small cercariae) with measurements of Jouet et al. [43].

Snail Host	*Radix peregra*	*Radix balthica*	*Radix balthica*	*Radix balthica*	*Radix balthica*	*Radix balthica*	*Radix balthica*
**Source**	Jouet et al. [43]	Present study	Present study	Present study	Present study	Present study	Present study
**Group of cercariae**	–	Large morphs ^1^	Small morphs ^1^	Other small cercariae ^2^	Large morphs ^1^	Small morphs ^1^	Other small cercariae ^2^
**Fixation method**	Live and formalin	Live	Live	Live	Hot formalin	Hot formalin	Hot formalin
**No. of cercariae measured**	31	7	8	23	9	16	42
Total length	864	1368	994	970	1339	944	881
Body length	257	340	276	272	278	241	223
Body width	73	110	90	93	75	66	62
Tail stem length	379	702	449	432	741	461	421
Tail stem width	50	62	57	52	54	47	44
Furca length	227	326	270	265	320	242	237
Head organ length	–	119	93	97	85	74	73
Head organ width	–	76	64	64	50	45	43
Diameter of acetabulum	28	34	27	34	29	26	25
Center of acetabulum to anterior body end	182	212	165	161	167	137	134
Diameter of eye spot	–	9	8	7	9	8	8
Eye spot to anterior body end	134	145	124	120	123	112	103
Body length/Body width	3.52 ^3^	3.08	3.07	2.96	3.76	3.70	3.71
Body length/Tail stem length	0.68 ^3^	0.49	0.62	0.65	0.38	0.52	0.54
Tail stem length/Furca length	1.69 ^3^	2.17	1.67	1.62	2.32	1.91	1.78

^1^ Two distinct size morphs of cercariae isolated from one snail individual, ^2^ Cercariae isolated from four snail individuals, and ^3^ Calculated from the mean values in the original article.

**Table 2:** Results of separate one-way ANOVA (analysis of variance) tests for two data sets (live and formalin-fixed specimens) evaluating the differences in morphometrical parameters of cercariae of *Trichobilharzia* sp. “peregra” among three groups of cercariae (large and small morphs, and other small cercariae). Statistically significant results (at α = 0.05) are indicated in bold. See Table 1 for the number of cercariae measured and entered into statistical analyses.

Data Set	Live Cercariae				Formalin-Fixed Cercariae			
Parameter Tested	Df ^1^	MS ^2^	F ^3^	*p* ^4^	Df	MS ^2^	F ^3^	*p* ^4^
Total length	2	0.348	17.84	**<0.001**	2	0.656	153.1	**<0.001**
Body length	2	0.138	9.13	**<0.001**	2	0.189	21.3	**<0.001**
Body width	2	0.095	9.18	**<0.001**	2	0.147	8.49	**<0.001**
Tail stem length	2	0.713	16.11	**<0.001**	2	1.210	115.7	**<0.001**
Tail stem width	2	0.106	3.14	0.056	2	0.129	13.30	**<0.001**
Furca length	2	0.123	8.63	**<0.001**	2	0.343	45.6	**<0.001**
Head organ length	2	0.137	18.17	**<0.001**	2	0.093	11.5	**<0.001**
Head organ width	2	0.086	12.27	**<0.001**	2	0.074	10.94	**<0.001**
Diameter of acetabulum	2	0.153	4.36	**<0.05**	2	0.075	6.17	**<0.01**
Center of acetabulum to anterior body end	2	0.216	9.52	**<0.001**	2	0.160	11.31	**<0.001**
Diameter of eye spot	2	0.093	13.55	**<0.001**	2	0.086	14.33	**<0.001**
Eye spot to anterior body end	2	0.101	5.99	**<0.01**	2	0.137	12.93	**<0.001**
Body length/Body width	2	0.005	0.57	0.570	2	0.001	0.06	0.94
Body length/Tail stem length	2	0.030	6.68	**<0.01**	2	0.046	17.39	**<0.001**
Tail stem length/Furca length	2	0.101	13.08	**<0.001**	2	0.121	32.25	**<0.001**

^1^ Degrees of freedom, ^2^ Means of squares, ^3^ Test criterion value, and ^4^ Level of significance.

## Data Availability

Sequence data reported in this paper are available in the GenBank under accession numbers OM716986, OM716987. Data supplemental to the main text are provided in Appendix A. Raw and processed data can be shared on reasonable personal request directly from the corresponding author.

## Appendix A

**Table A1:** Comparative metrical data for live specimens of cercariae of *Trichobilharzia* sp. “peregra” isolated from naturally infected *Radix balthica* snails. Data are presented in micrometers as the mean ± SD and range (minimum to maximum values). The width values correspond to maximum width measurements.

Group of Cercariae	Large Morphs ^1^		Small Morphs ^1^		Other Small Cercariae ^2^	
No. of Cercariae Measured	7		8		23	
	Mean ± SD	Range	Mean ± SD	Range	Mean ± SD	Range
Total length	1368 ± 49	1302–1420	994 ± 65	901–1067	970 ± 157	668–1208
Body length	340 ± 42	304–427	276 ± 20	242–305	272 ± 36	200–332
Body width	110 ± 11	94–127	90 ± 4	85–97	93 ± 10	63–105
Tail stem length	702 ± 41	621–745	449 ± 39	401–497	432 ± 99	241–564
Tail stem width	62 ± 6	54–70	57 ± 4	50–62	52 ± 10	22–68
Furca length	326 ± 28	276–353	270 ± 23	236–292	265 ± 34	184–322
Head organ length	119 ± 9	105–130	93 ± 4	90–98	97 ± 9	72–108
Head organ width	76 ± 7	65–84	64 ± 2	61–66	64 ± 6	52–80
Diameter of acetabulum	34 ± 9	26–48	27 ± 7	21–39	34 ± 5	22–43
Center of acetabulum to anterior body end	212 ± 29	178–260	165 ± 17	141–191	161 ± 27	120–215
Diameter of eye spot	9 ± 1	8–10	8 ± 1	7–9	7 ± 1	6–9
Eye spot to anterior body end	145 ± 15	123–174	124 ± 13	111–151	120 ± 17	81–160
Body length/Body width	3.08	2.81–3.36	3.07	2.69–3.47	2.96	2.27–3.86
Body length/Tail stem length	0.49	0.43–0.60	0.62	0.53–0.76	0.65	0.49–1.01
Tail stem length/Furca length	2.17	1.84–2.60	1.67	1.57–1.88	1.62	0.97–1.91

^1^ Two distinct size morphs of cercariae isolated from one snail individual, and ^2^ Cercariae isolated from four snail individuals.

**Table A2:** Comparative metrical data for formalin-fixed specimens of cercariae of *Trichobilharzia* sp. “peregra” isolated from naturally infected *Radix balthica* snails. Data are presented in micrometers as the mean ± SD and range (minimum to maximum values). The width values correspond to maximum measurements.

Group of Cercariae	Large Morphs ^1^		Small Morphs ^1^		Other Small Cercariae ^2^	
No. of Cercariae Measured	9		16		42	
	Mean ± SD	Range	Mean ± SD	Range	Mean ± SD	Range
Total length	1339 ± 51	1270–1436	944 ± 37	874–1023	881 ± 66	733–991
Body length	278 ± 25	241–317	241 ± 30	201–332	223 ± 19	190–266
Body width	75 ± 10	65–87	66 ± 6	54–75	62 ± 9	45–78
Tail stem length	741 ± 39	680–789	461 ± 14	426–481	421 ± 50	320–499
Tail stem width	54 ± 5	49–64	47 ± 4	41–56	44 ± 5	34–54
Furca length	320 ± 17	294–340	242 ± 15	217–263	237 ± 22	169–281
Head organ length	85 ± 7	72–94	74 ± 8	62–91	73 ± 6	59–84
Head organ width	50 ± 6	42–57	45 ± 2	42–50	43 ± 4	38–55
Diameter of acetabulum	29 ± 2	26–33	26 ± 3	20–31	25 ± 3	20–33
Center of acetabulum to anterior body end	167 ± 29	105–196	137 ± 17	108–168	134 ± 13	107–160
Diameter of eye spot	9 ± 1	8–11	8 ± 1	7–9	8 ± 1	7–9
Eye spot to anterior body end	123 ± 14	104–148	112 ± 9	93–130	103 ± 11	80–126
Body length/Body width	3.76	2.97–4.65	3.70	2.91–5.19	3.71	2.56–5.56
Body length/Tail stem length	0.38	0.31–0.45	0.52	0.45–0.74	0.54	0.41–0.77
Tail stem length/Furca length	2.32	2.12–2.49	1.91	1.77–2.11	1.78	1.45–2.20

^1^ Two distinct size morphs of cercariae isolated from one snail individual, and ^2^ Cercariae isolated from four snail individuals.
